# Isolated Femoral Shaft Fracture in Wakeboarding and Review of the Literature

**DOI:** 10.1155/2020/8841395

**Published:** 2020-09-18

**Authors:** Henrik Constantin Bäcker, Seth Shoap, Gabor Vasarhelyi, Gergely Pánics

**Affiliations:** ^1^Department of Orthopaedics, Uzsoki Hospital, Mexikói Street 62, Budapest, Hungary; ^2^Charité – Universiätsmedizin Berlin, Corporate Member of Freie Universitat Berlin, Humboldt-Universitat zu Berlin, Berlin Institute of Health, Chariteplatz 1, Berlin, Germany; ^3^Columbia University Medical Center, 622 West 168th Street, 10032 New York, USA

## Abstract

**Introduction:**

Wakeboarding is an extreme sport that has shown increasing popularity in recent years, with an estimated 2.9 million participants in 2017. Due to this trend, injuries related to this sport are likely to become more common. Isolated femoral shaft are rare; however, they occur much more frequently in youth as a result of high velocity events, such as dashboard-related injuries. Few studies have addressed injuries related to wakeboarding, and of those that have, most have reported on muscle injuries, ligament ruptures, and sprains. Due to the dearth in literature, we want to present two cases of isolated noncontact femoral shaft fractures that resulted from wakeboarding. *Case Presentation*. Two 28-year-old, otherwise healthy, wakeboarders—patient A, male, and patient B, female—presented to our Department of Orthopaedics and Sports Medicine with isolated femoral shaft fractures. Both were admitted due to wakeboard-related noncontact injuries, where patient A fell while performing a sit-down start during cable wakeboarding and patient B after attempting a wake-jump. Both patients were being pulled by motorboats at roughly 40 km/h. After clinical examination and radiography, left spiral (AO classification: 32-A1.2) (patient A) and right-sided bending, wedge (AO classification 32-B2.2) (patient B) isolated femoral shaft fractures were diagnosed. No concomitant injuries were reported. For treatment, long reamed locked nails were applied, while the patients were under spinal anaesthesia. Physiotherapy was prescribed postoperatively. Patient A returned to wakeboarding 155 days after the surgery, and patient B returned after approximately half a year.

**Conclusion:**

This case series shows that even in noncontact sports such as wakeboarding, high-energy forces applied to the femur can cause isolated femoral shaft fractures. Despite multiple reports in various sports of stress fractures of the femur, there are few publications of direct trauma.

## 1. Introduction

Water sports including kitesurf, water ski, and wakeboarding have gained increasing popularity in the recent years since the development of wakesurfing in 1964 [1]. Typically, both of the athlete's feet are either fixed rigidly or with two slings to the board and pulled either from a boat or cable railway at approximately 28-39 km/h. In 2011, cable wakeboarding was even nominated as sports activity for the 2020 Olympic Games [2]. To allow for a more consistent pull and ultimately smoother ride, newer equipment was developed including more curved and compact boards and polypropylene ropes that are more stretch resistant [3]. The most common injury pattern during wakeboarding is when the tip of the wakeboard submerges into the water, leading to a canting of the board. Hereby, the lever arm forces are exerted on the ankle, knee ligaments, and muscles, leading to anterior cruciate ligament, collateral ligament, and hamstring tears [4]. In most cases, athletes release the handle to brace for the impact with the water, and ultimately avoid any severe injuries. However, as a precaution, safety equipment is highly recommended, including wearing a water-resistant helmet, newer ropes, and life vests [5].

Despite the rarity of isolated femoral shaft fractures, they are found much more frequently in youth between 2 and 3 and a second peak between 17 and 18 years old. Fractures in this demographic are typically the result of high-energy trauma [6]. The incidence of femoral fractures is on the rise as a result of increased participation in organized sports and extracurricular activities. According to a nationwide study, femoral fractures account for 21.7% of hospital admissions for traumatic orthopaedic injuries [7].

Despite reports of femoral stress fractures in various sports, the literature focusing on direct trauma in wakeboarding is very scarce. Therefore, our purpose is to illustrate two cases of femoral shaft fractures in recreational wakeboarders and to review the pertinent literature. Moreover, this study aims to illustrate the possible dynamics of injuries which may be specific of this sport. We hypothesized that improper technique when performing a sit-down start or a wake jump can lead to high twisting forces. This may lead to a submersion of the wakeboard in the water while performing either a jump or a sit-down start.

## 2. Case Presentation

**Figure 1:**
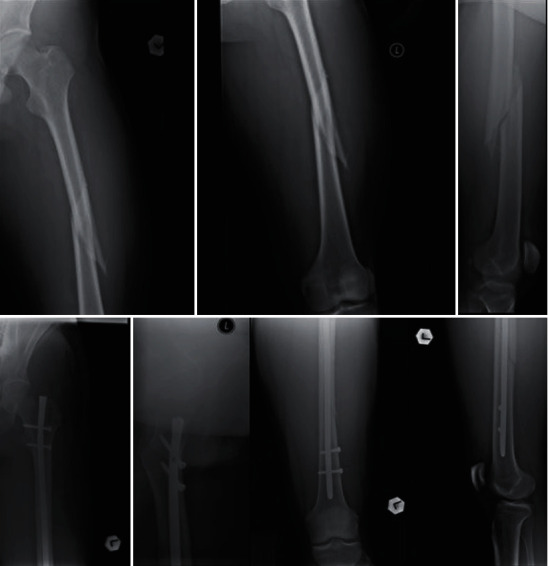
Patient A, a 28-year-old male patient with left-sided midshaft spiral femoral fracture before and after surgery.

**Figure 2:**
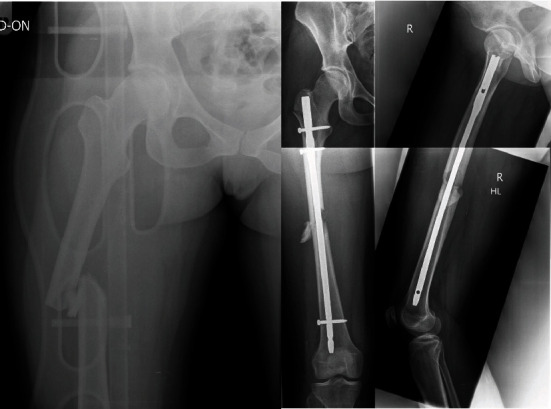
Patient B, a 28-year-old female patient with right-sided midshaft femoral fracture before and after surgery.

Patient A, a 28-year-old, otherwise-healthy, male recreational regular-footed (right foot forward) wakeboarder made a sit-down start from a starting ramp in a cable park. When he did so, the tip of his wakeboard sank in the water, and the board rotated, along with both of his attached lower limbs. This resulted in a violent twisting force to his left pelvis and thigh. He collapsed into the water, and simultaneously felt a sharp pain in his left thigh. He was rescued from the water immediately by the staff of the cable park and attended to by paramedics at the hospital around 20 minutes after the accident. Upon arrival in the hospital, a spiral femur shaft fracture (AO classification: 32-A1.2) was confirmed on radiographs (Figure 1); no motor or sensory disabilities were identified. There was swelling and crepitation in the mid-third of the left thigh. After a short preoperative preparation, anterograde reamed locked nailing was performed using a rigid locked nail while the patient was under spinal anaesthesia (Figure 2). He had no postoperative complications and recovered well through a physical therapy program. At 6 months follow-up, the patient had full, painless ROM, with a little muscle atrophy in the injured thigh. He returned to wakeboarding 155 days after injury.

Patient B, a 28-year-old, otherwise healthy, female, advanced, goofy-footed (left foot forward) wakeboarder tried to perform a wake jump. As she came back down upon the surface of the water, she fell on her right leg. No solid object, such as a tree branch or rock, was reported to have been in the water. Similar to patient A, we assume that during the landing, the tip of the wakeboard submerged into the water and was immediately followed by a rotation of the board, causing a twisting force on her right pelvis and thigh. Moreover, as the athlete was prepared for high forces, she contracted all muscles, particularly, of the knee and ankle, for a maximum of stability. When she arrived in the hospital, she presented with swelling and crepitation in the second third of the right femur. A wedge, bending, femoral shaft fracture (AO classification: 32-B2.2) was diagnosed after X-ray examination. Similar to patient A, after a short preoperative preparation, an anterograde reamed locked nailing was performed under a rigid lock nail in spinal aesthesia (Figure 2). No postoperative problems could be observed, and she recovered well and quick through a physical therapy program.

**Figure 3:**
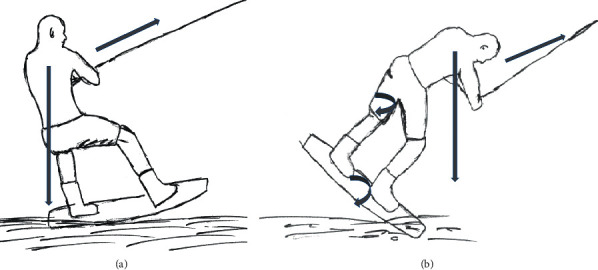
(a) Normal wakeboard ride with forward pulling forces and weightbearing of the backford foot (goofy footed); (b) submerge front tip of the wakeboard with pulling forces to the front and nearly fully extended back foot leg with rotational forces of the wakeboard and subsequently of the back foot femur.

It is likely that in both cases, similar dynamics caused this type of injury. Typically, the forward foot is slightly more bent in order to lift the tip of the board out of the water. Once the tip sinks in the water, the board submerges, and the maximum forces applied to the back foot (standing foot) which is nearly fully extended. Hereby, the highest stress is forwarded onto the femoral shaft, leading to an isolated fracture (Figure 3).

## 3. Discussion

There is a dearth in literature on wakeboarding and isolated femoral diaphyseal fractures. Femoral diaphyseal fractures are reported in 10 out of 100,000 people each year, and the incidence rate ratio between men and women is 0.9 (*p* < 0.001) [8]. The overall incidence of sport-related femoral fractures in athletes is 0.12% per year. Rewers et al. reported that sports are responsible for more than one-fifth of all femoral fractures in the young population [9]. Among the athletic population, numerous studies have been performed evaluating stress fractures of the femur. According to their results, this type of injury is relatively uncommon, and data from the literature suggest that they constitute only 2.8–7% of all sport-related stress fractures [10–12].

In wakeboarding, the overall incidence of injuries ranges from 1.32/1,000 hours (or 1.41/1000 runs) to 12/1,000 hours, of which most (61%) are considered mild injuries [13–15]. The most common injury types include laceration, ligament rupture, and strain. The fracture incidence is reported to be 15%, with the most frequently injured body parts being the head and knee [13, 15]. There is only one paper that reported a clinical case of selective femur fractures among wakeboarders [16]. Schofer et al. evaluated the possible injury mechanism in cable wakeboarding and reported that only 2% of injuries occur at the start of a run [13].

A comparable sport to wakeboarding with similar energetic impact is kitesurfing. Here, the incidence of injuries is similar, ranging from 1.04/1,000 h to 18.5/1,000 h in competitive athletes, which is higher than in windsurfing; however, lower than in other sports such as motocross, soccer, and American football [17–20]. Among all injured athletes, only 4.0% (*n* = 7/177) suffered from fractures (lumbar spine, bimalleolar ankle, and wrist fractures). None of the fractures occurred while attempting a jump. Hereby, no femoral fractures were observed and reported. The most common injuries were cuts and abrasions, followed by contusions and joint or muscle sprains. Furthermore, the reported injuries were a result of patients losing control of their kite in 10.7% or gear failure in 3.4% [21]. The authors hypothesized that the feet and ankles are especially at risk during landing since the forefoot is fixed in straps during rotational manoeuvres. The injury mechanism in aquatic sports, especially in water skiing, showed a correlation by level of experience, as beginners are injured more frequently while submerged during take-off, with douche and enema injuries, while experts most commonly injured their knees, backs, shoulders, hand, and foot while falling [14, 22].

In wakeboarding, an athlete requires a relatively high degree of strength in the legs and core in order to be stabilized, especially during landing and starting, where the maximal forces are exerted. Once the front tip of the wakeboard submerges into the water, the forefoot is released from the strap, and the torsional forces are extracted onto the rear standing foot. These forces are exerted bimodally, from both the pulling forces of the cable or motorboat, as well as the rotation forces when the board turns to the side as shown in Figure 3. Sallay et al. found a similar injury pattern of the thigh among novice water skiers [23]. The skiers were injured as they attempted to a submerged water take-off on one or two skis. In each case, the skiers attempted to immediately stand up on their skis by extending their knees as the boat began its acceleration. This posture caused the ski tips to submerge beneath the surface of the water, resulting in sudden deceleration of the lower extremities. The forward momentum from the tow rope, attached to the accelerating boat rapidly pulled the trunk forward, causing extreme hip flexion. Therefore, athletes who tense all their muscles and are not familiar with the forward momentum from the tow rope are more at risk, according to this theory. This may also explain why patients who are not completely stiff through muscle tension, such as with slightly bended knees, are more prone to suffering from soft tissue injuries, including ankle, collateral, and cruciate ligament tears [4].

Since the lower leg consists of the proximal and distal tibiofibular joints, there is some degree of shock absorption, whereas the femur is rigid. Sports in which femoral fractures are more common are typically a result of high-energy activities. This includes motocross, where 10% of injuries are femur fractures [24] and alpine skiing [25]. In addition, correlational studies have found that sport-related femoral fractures are more likely to occur during winter sports, such as skiing and snowboarding, or summer sports such as football [9, 26, 27]. In equestrian-related injuries, 1.8% of all fractures involved the femur [28]. In skiing, the incidence is around 15%, with the leading mechanism of injury a collision or a fall with high speed [29]. In skateboarding, 6.1% of all injuries were femoral fractures—with the younger age group (below the age of 10 years) being more affected [30]. In contrast, in snowboarding, the prevalence is almost 2-3 times higher with 9.5%-14.9% [31, 32]. In aerial sports, such as skydiving, paragliding, and BASE jumping, 15 femoral fractures were observed in 122 patients who suffered from fractures (12.3%). The femoral shaft was specifically affected in 12 of these patients (*n* = 12/15; 80.0%). These fractures resulted from direct traumas, and many of these patients suffered from other concomitant injuries. Hereby, the overall mean injury severity score was 16.5 (SD 14.0), and the most commonly observed fractured body part is the lumbar spine [33]. In regard to skydiving, the lower extremity is primarily affected, with 51% of cases and 13 thigh fractures out the 160 total injuries of the lower extremity [34].

In extreme sports, such as kitesurfing, sharp accelerations and their resulting forces acting on the body can cause these types of injuries. This was reported by Spanjersberg and Schipper who reported on one patient who was picked up by a strong wind after surfing too close to the shore. The surfer lost his kite and was thrown over a dyke, landing directly on top of a parked car, which resulted in a femur fracture, shoulder dislocation, and face laceration [35]. Therefore, to avoid these devastating injuries, especially in these more extreme sports, extensive coaching should be performed in order to thoroughly practice these manoeuvres. This may include changing and safe guarding certain wakeboarding manoeuvers, sport-specific training, or designing apparel that can better distribute these high-energy forces.

## 4. Conclusion

We report two cases of noncontact femoral shaft fractures in an amateur and advanced wakeboarder. Despite one previous report of this injury in water skiing, these are the first reported cases in both closed-course cable wakeboarding and wakeboarding involving a wake jump involving a motor vehicle. Coaches should take extra measures when teaching novice wakeboarders a proper starting technique and proper tricks and jump techniques in advanced athletes.

## 5. Clinical Message

This study shows that isolated noncontact femoral shaft fractures can occur during a sit-down start and wake jump. Proper technique is required to prevent severe injuries. Wakeboarders should be trained to prevent any complications.

## Data Availability

The data used to support the findings of this study are included within the article.
